# Ultrasonic emissions during ice nucleation and propagation in plant xylem

**DOI:** 10.1111/nph.13361

**Published:** 2015-03-10

**Authors:** Guillaume Charrier, Manuel Pramsohler, Katline Charra‐Vaskou, Marc Saudreau, Thierry Améglio, Gilbert Neuner, Stefan Mayr

**Affiliations:** ^1^ Department of BotanyUniversity of InnsbruckSternwartestrasse. 15A‐6020InnsbruckAustria; ^2^ INRAUMR547 PIAFF‐63100Clermont‐FerrandFrance; ^3^ Clermont UniversitéUniversité Blaise PascalUMR547 PIAFF‐63100Clermont‐FerrandFrance

**Keywords:** cavitation, freezing stress, ice nucleation, ice propagation, infrared thermography, nondestructive monitoring, ultrasonic acoustic emissions, xylem

## Abstract

- Ultrasonic acoustic emission analysis enables nondestructive monitoring of damage in dehydrating or freezing plant xylem.
- We studied acoustic emissions (AE) in freezing stems during ice nucleation and propagation, by combining acoustic and infrared thermography techniques and controlling the ice nucleation point.
- Ultrasonic activity in freezing samples of *Picea abies* showed two distinct phases: the first on ice nucleation and propagation (up to 50 AE s^−1^; reversely proportional to the distance to ice nucleation point), and the second (up to 2.5 AE s^−1^) after dissipation of the exothermal heat. Identical patterns were observed in other conifer and angiosperm species.
- The complex AE patterns are explained by the low water potential of ice at the ice–liquid interface, which induced numerous and strong signals. Ice propagation velocities were estimated via AE (during the first phase) and infrared thermography. Acoustic activity ceased before the second phase probably because the exothermal heating and the volume expansion of ice caused decreasing tensions. Results indicate cavitation events at the ice front leading to AE. Ultrasonic emission analysis enabled new insights into the complex process of xylem freezing and might be used to monitor ice propagation *in natura*.

## Introduction

Among the abiotic factors driving plant distribution, frost is critical in several environments, such as temperate, boreal and alpine areas. Freezing can damage plants based on mechanic or hydraulic mechanisms. Mechanical constraints, induced by the volume increase due to the water–ice transition, can generate frost cracks (Ishida, 1963; Cinotti, 1991) and impact on subsequent drought resistance (Charra‐Vaskou *et al*., 2012; Christensen‐Dalsgaard & Tyree, 2013, 2014). Intracellular ice formation, whenever it occurs, is lethal for the cell (Wolfe & Bryant, 2001). The low water potential at the ice–liquid interface (Ψ_ice_) can induce severe osmotic stress and plasmolysis of cells (Steponkus, 1981; Ruelland *et al*., 2009; Charrier *et al*., 2013a), and freeze–thaw cycles can lead to embolism in xylem conduits (Sperry & Sullivan, 1992; Tyree *et al*., 1994; Hacke *et al*., 2001; Mayr *et al*., 2007, 2014). Accordingly, resistance to winter embolism resulting from freeze–thaw cycles has been identified as a critical trait in tree species (Charrier *et al*., 2013b).

During freezing of conduits, dissolved gases are entrapped in bubbles within the forming ice because the solubility of gas is lower in ice than in liquid (Sevanto *et al*., 2012). At thawing, these bubbles can redissolve unless tension forces their expansion (Lemoine *et al*., 1999; Hacke & Sperry, 2001; Cruiziat *et al*., 2002; Tyree & Zimmermann, 2002). Wider conduits contain more gas, causing larger bubbles, which expand at lower negative tension. Conduit diameter and xylem sap tension are therefore critical for the formation of freeze–thaw induced embolism (Davis *et al*., 1999; Pittermann & Sperry, 2003; Charrier *et al*., 2013b). Accordingly, Mayr & Sperry (2010) demonstrated that bubbles expand during thawing when under sufficient tension.

Acoustic emissions (AEs) analysis has been extensively proven for monitoring drought‐induced embolism (Tyree & Dixon, 1983; Salleo & Lo Gullo, 1986; Mayr & Rosner, 2011; Ponomarenko *et al*., 2014). During drought, the xylem tension increases until the sap turns into vapour (cavitation), which releases acoustic energy, recordable as AEs. In wood, AEs were also observed after the onset of freezing (Raschi *et al*., 1989; Kikuta & Richter, 2003; Mayr *et al*., 2007; Mayr & Zublasing, 2010). It was suggested that the low Ψ_ice_ at the ice–liquid interface attracts the water molecules toward the ice lattice and thus increases the tension into the liquid. When the critical threshold for cavitation is reached, it causes bubble formation in the adjacent sap. AEs may thus be generated by cavitation in the wood of conifers due to low Ψ_ice_, increasing the tension until vulnerability thresholds are reached (Mayr *et al*., 2007). Lower temperatures were found to induce more AEs corresponding to the strong temperature‐dependence of Ψ_ice_ (Charrier *et al*., 2014a).

Ice formation in wood is a dynamic process: when the temperature is negative, water remains liquid in a metastable state (supercooling) until it turns into ice around a nucleus. Such nucleii might be bacteria, impurities, cell walls (heterogeneous nucleation) or even water molecules themselves when temperatures fall below −38°C (homogeneous nucleation). After nucleation, water molecules crystallize around the nucleus and ice propagates longitudinally and radially within the xylem (Neuner *et al*., 2010). Two different mechanisms thereby generate water fluxes: the low water potential (Ψ) pulls water toward the ice–liquid interface (Améglio *et al*., 2001), whereas the increased volume of ice (+ 10% compared with liquid water) induces positive pressure.

The process is further complicated as ice formation is an exothermal process diminishing the temperature decrease and solutes are concentrated in the liquid phase, increasing tensions in the remaining sap. Freezing normally starts and occurs in the apoplast, whereas intracellular sap usually freezes at lower temperature. Recently, freezing was demonstrated to induce a relevant flow of CO_2_ out of stems, which leads to substantial changes in gas concentrations within the sap (Lintunen *et al*., 2014). Freezing in wood is not well understood and, up to now, AEs have not been related to the spatial and dynamic patterns of ice formation in wood. In this study, we analysed AEs and ice formation dynamics in freezing branches according to the following hypotheses: the origin of AEs is located at the front of the growing ice; in consequence, the source of AE moves with the ice expansion; and the degree of supercooling (∆*T*) influences AE patterns.

We studied freezing in branches via thermocouples and infrared thermography, and recorded AEs by several sensors mounted at different positions along samples. Experiments were performed under controlled conditions in freezing chambers, which enabled comparison of spontaneous and induced ice nucleation, species‐specific freezing and AE patterns, as well as patterns at different ∆*T*.

## Materials and Methods

### Plant material

Branches, 1–1.5‐m long, were sampled from mature *Picea abies* (L.) H. Karst growing near the Department of Botany in Innsbruck, Austria, in April 2013, before budburst and resumption of cambial growth. Tension in samples was released overnight, the basal end in clear water and wrapped in a plastic bag at 5°C. Branches were bench‐dehydrated until they reached a Ψ of −2.8 MPa corresponding to 12% loss of conductivity: (Ψ_12_; supplement Choat *et al*., 2012). This critical tension is known to generate many AEs (Mayr & Zublasing, 2010). Water potential was measured on end twigs using a Scholander pressure chamber (model 1000 Pressure Chamber; PMS Instrument Company, Albany, OR, USA). In addition, two other angiosperms and conifers were harvested and dehydrated to their respective Ψ_12_ values: *Abies alba* Mill., 1759 (−3.3 MPa), *Carpinus betulus* L., 1753 (−3.2 MPa), *Corylus avellana* L., 1753 (−2.0 MPa) and *Pinus mugo* Turra, 1764 (−3.5 MPa).

Samples, *c*. 360 mm in length and 10–20 mm in diameter, were cut from the main stem of branches and covered with parafilm (Alcan, Montreal, QC, Canada) to avoid further dehydration. A small area (*c*. 25 mm²) was debarked for ice nucleation of the sample at the sap wood and covered by a removable piece of parafilm.

**Figure 1:**
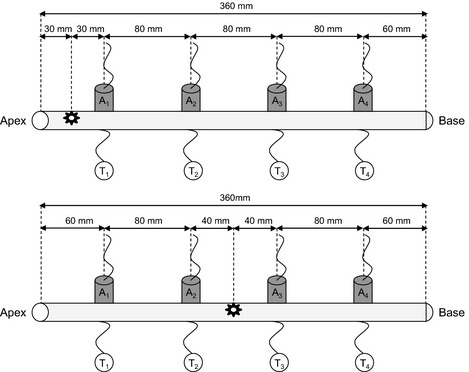
Experimental set‐up used in the study. Acoustic sensors (A_1_–A_4_) and thermocouples (T_1_–T_4_) were clamped every 80 mm along the samples (branches from *Picea abies*,* Abies alba*,* Pinus mugo*,* Carpinus betulus* or *Corylus avellana*). The sites of artificial ice nucleation are indicated with stars (at the apical end for all species, upper panel, or in the centre only for *Picea abies*, lower panel).

The main experimental set‐up (Fig. 1) was conducted on five branches in *P. abies* and three branches in *A. alba*,* C. betulus*,* C. avellana* and *P. mugo*. The effect of initial conditions was tested independently on three branches of *P. abies* (Ψ *c*. −1.0 MPa; bark removal; parafilm insulation; nucleation point located in the centre of the sample; spontaneous ice nucleation; degree of supercooling from 2.7 to 6.8 K).

### AE

Ultrasonic measurements were performed with a PCI‐8‐based system (PAC12518‐bitA/D, 20 kHz–1 MHz) and 150 kHz resonance sensors (R15) connected to a preamplifier set to 40 dB (all components: Physical Acoustics, Wolfegg, Germany). The threshold was set to 45 dB, the gain to 40 dB (Mayr *et al*., 2007; Mayr & Rosner, 2011). Registration and analysis of ultrasonic events were performed with AEwin software (Mistras Holdings Corp., Princeton, NJ, USA). About 100 mm² of parafilm and bark were removed and the xylem was covered with silicone grease (to ensure sufficient acoustic coupling and prevent dehydration) before attaching the sensors with clamps. Two sensors were placed at a distance of 60 mm and two at 140 mm from the sample ends (Fig. 1). Coupling was tested with lead breaks (Hsu Nielsen method NF EN1330‐9; Charrier *et al*., 2014b), and the sensors were reinstalled in case the amplitude of the signal was < 90 dB. Propagation of the AEs source during phase I (see the Results section for description) was estimated as the distance between two acoustic sensors divided by the difference in time of maximal AE activity.

### Freeze–thaw cycles

Freeze–thaw cycles were performed in a computer‐controlled freezer (described in Hacker & Neuner, 2007). Xylem temperatures were monitored using copper‐constantan thermocouples inserted through the bark, in contact with xylem, at the opposite side of the acoustic sensors. Air temperature was monitored by a copper‐constantan thermocouple in the middle of the chamber and used to control the temperature changes within the chamber. Temperature values were recorded every 4 s, which was the shortest interval possible. The temperature was stabilized at + 2°C for at least 30 min before cooling at a rate of 5 K h^−1^. A minimal temperature of −10°C was kept for 4 h before thawing at a rate of 5 K h^−1^ to + 2°C.

### Controlled ice nucleation of the sample

When the temperature in the freezer reached the desired level, the small piece of parafilm on the debarked area was quickly removed by hand. After a few minutes for temperature stabilization, a piece of ice, stored at −20°C, was fixed in a clamp attached to a 1‐m‐long metal stick and put in contact with the wood until an exotherm was recorded by the thermocouples in the wood.

### Infrared thermography

Ice propagation was also monitored by use of a digital infrared camera (ThermaCAM S60; FLIR Systems AB, Danderyd, Sweden). The camera was placed in an isolated box at the top of the freezing chamber and infrared images of the experimental set‐up were recorded at 100‐ms time resolution. Infrared images obtained during freezing of the samples were analysed by infrared differential thermal analysis (IDTA; Hacker & Neuner, 2007). IDTA images show surface temperature changes, that is, the latent heat released during freezing of water in samples. The software ThermaCAM Researcher (FLIR Systems AB) was used to control the infrared camera and to analyse images. Ice propagation velocity was estimated with infrared pictures as the distance between two acoustic sensors divided by the duration of the exothermal signal to propagate from one sensor to the other.

### Statistical analyses

We calculated linear regression and *P*‐values, after testing the normality of the distribution with Shapiro–Wilk test and homogeneity of variances with *F*‐test, using R software (R Development Core Team, 2005). Significant differences between slopes of linear regression were tested with *t*‐test. For non‐normal distribution, a Kruskal–Wallis test was used.

## Results

### Ice nucleation and propagation in *Picea abies*

**Figure 2:**
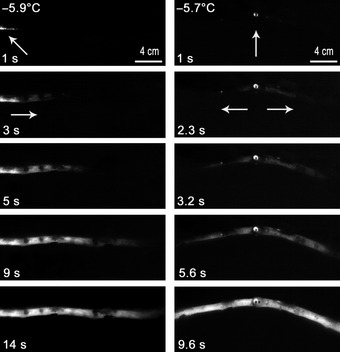
Typical ice propagation monitored by infrared thermography in branches from *Picea abies* after artificial ice nucleation at one end (left column) or in the centre of the sample (right column). Arrows indicate ice nucleation points and directions of ice propagation. In the image sequence, the temperature at the time of nucleation and the time after nucleation(s) are given.

**Figure 3:**
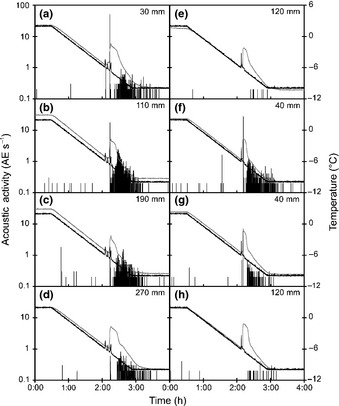
Typical acoustic activity (acoustic emissions s^−1^) in the wood of *Picea abies* at different sensors during freezing. Sensors were positioned as in Fig. 1, and data are from the same experiment as in Fig. 2. When ice nucleation was induced at one end (a–d), sensors were (a) 30 mm, (b) 110 mm, (c) 190 mm and (d) 270 mm from the nucleation point. When nucleation was induced in the centre (e–h), sensors were (f, g) 40 mm and (e, h) 120 mm from the nucleation point. Black and grey lines represent air and xylem temperatures.

Sample temperature, relative to air temperature, locally increased when ice nucleation was induced near to the end as well as in the middle of the sample (Fig. 2). In consequence, the freezing exotherm propagated along the sample within *c*. 20 s, and the difference to the air temperature was visible for *c*. 20 min. Infrared pictures were consistent with thermocouple measurements as the sample temperature suddenly increased from −6 to −1°C when the sample started to freeze (Fig. 3). Temperatures stabilized between −1 and −2°C for *c*. 15 min before they progressively decreased to air temperature.

At all acoustic sensors, AE activity (AE s^−1^) was almost negligible (< 1–2 AE s^−1^) before ice nucleation occurred. On ice nucleation, we observed a two‐phased spatial and dynamic pattern of AEs (Table 1). On phase I of the acoustic activity (during *c*. 20 s), sensors close to the nucleation point revealed highest AE activities immediately after the exotherm indicated freezing. On one end of the sample (30 mm from the nucleation point), *c*. 540 AEs were recorded with a peak activity of 49.8 AE s^−1^ (Fig. 3a). Along the sample, peak activity decreased to 17.5 at 110 mm (Fig. 3b), 1.8 at 190 mm (Fig. 3c) and 0.6 AE s^−1^ at 270 mm distance (Fig. 3d). A similar pattern was observed when ice nucleated in the centre of the sample (Fig. 3e–h). We observed 7 and 26.8 AE s^−1^ at 40 mm distance from the nucleation point (Fig. 3f,g), whereas no AEs were recorded at 120 mm distance (Fig. 3e,h). After the first peak in AE activity, AE activity ceased (< 0.5 AE s^−1^ on all sensors), but increased again after *c*. 20 min (phase II): maximum rates then were 0.9–2.5 AE s^−1^. Acoustic activity during phase II was similar on all sensors. However, we observed two‐fold more AEs in the centre of the sample (mean ± SE = 885 ± 222) than on the ends (mean ± SE = 358 ± 143) for both positions of ice nucleation (Fig. 3).

**Table 1:** Percentage of acoustic emissions (Means ± SE; *n *=* *5 replicates) detected during the different phases depending on the distance from the ice nucleation point in the wood of *Picea abies*

Phase	Distance			
	30 mm	110 mm	190 mm	270 mm
Before freezing	7.6 ± 2.1%^a^	6.5 ± 0.7%^a^	8.1 ± 3.3%^a^	9.1 ± 3.0%^a^
I	45.6 ± 8.5%^a^	12.4 ± 8.8%^b^	2.1 ± 1.7%^b^	0.4 ± 0.1%^b^
II	46.8 ± 6.9%^a^	81.2 ± 8.4%^b^	89.8 ± 4.8%^b^	90.5 ± 2.9%^b^

Different letters indicate significant differences between distances.

**Figure 4:**
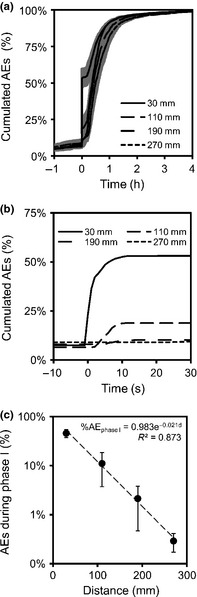
(a) Mean cumulated number of acoustic signals s^−1^ in the wood of *Picea abies* depending on the time of ice nucleation (*x*‐axis) and distance from the nucleation point (30, 110, 190 and 270 mm), ice nucleation at one end of the sample (black lines and grey areas represent mean and ±SE from five replicates, respectively). (b) Zoom from 10 s before to 30 s after ice nucleation from the previous graphic. SEs were removed for clarity. (c) Proportion of acoustic emissions (AEs) during phase I (from 1 s before to 30 s after ice nucleation) depending on the distance from the ice nucleation point.

Across replicates, the pattern of cumulated AEs was highly reproducible and depended on the distance from the ice nucleation point when nucleation occurred at one end (Fig. 4a) or in the centre (data not shown). Before freezing, only minor AE activities were recorded independent of the distance (from 6.5 to 9.1% of total AEs). During phase I, 46% of AEs were generated within a short period (< 10 s; 10–50 AE s^−1^) close to the ice nucleation point (Fig. 4b). Fewer AEs were recorded with increasing distance from the nucleation point (12.4, 2.1 and 0.4% at 110, 190 and 270 mm, respectively; Table 1). No AEs were recorded during the following *c*. 20 min until the sample temperature proceeded to decrease. Then, during phase II, most AEs were recorded within *c*. 45 min (*c*. 1–2 AE s^−1^). A strong exponential relationship was found between the proportion of AEs observed during phase I and distance to ice nucleation (*d*) (% AE_phaseI_ = 0.98·e^−0.021·*d*^; *R*² = 0.873; *P *<* *0.001; Fig. 4c).

### Effect of ice nucleation conditions in *P. abies*

**Figure 5:**
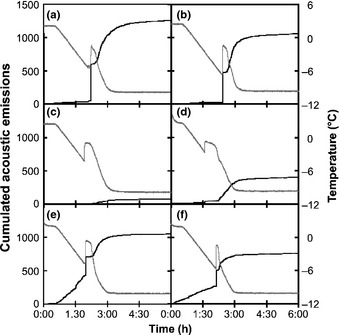
Typical dynamic of temperature (grey lines) and cumulated ultrasonic emissions (sensor close to the ice nucleation point, black lines) in the wood of *Picea abies*: standard experimental design as in Fig. 2 with ice nucleation at −6°C and Ψ = −2.8 MPa (a), spontaneous nucleation (b), Ψ = −1.0 MPa (c), temperature of nucleation = −2°C (d), sample without bark (e) and sample without parafilm (f).

**Figure 6:**
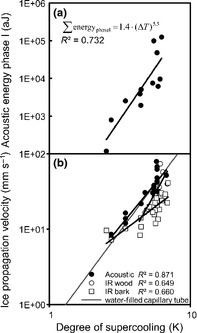
(a) Cumulative acoustic energy during phase I (30 mm from the nucleation point) depending on the degree of supercooling in the wood of *Picea abies* nucleated with ice. (b) Ice propagation velocity determined by acoustic emissions, or infrared thermography through bark or directly on wood (in debarked samples) in relation to the degree of supercooling, compared to the ice propagation velocity in a water‐filled capillary (grey line).

Artificially induced ice nucleation caused AE patterns similar to spontaneous nucleation (at similar ∆*T*). Similar patterns were also observed in debarked samples or samples not covered with parafilm (Fig. 5). Samples which were not sufficiently dehydrated (Ψ = −1.0 MPa) did not produce AE before or during ice nucleation and only few AEs (< 100) after ice nucleation (Fig. 5c). When samples were nucleated at lower ∆*T* (2–3 K), phase I showed only few AEs (*c*. 10 AEs), whereas phase II was similar to nucleation at −6°C. Accordingly, we observed a strong power law relation between the energy of AEs recorded during phase I and ∆*T* (∑ energy_phaseI_ = 1.4·(Δ*T*)^5.5^; *R*² = 0.732; *P *<* *0.001; Fig. 6a). The velocities of propagation of AEs during phase I showed a strong power law relation with ∆*T* (*V* = 0.78 · (Δ*T*)^2.28^; *R*² = 0.871; *P *<* *0.001; Fig. 6b) with similar slope (*P *=* *0.234) to velocities observed in debarked samples by infrared thermography (*V* = 0.09·(Δ*T*)^3.34^; *R*² = 0.649; *P *=* *0.034). By contrast, infrared analyses in intact samples showed slightly lower velocities (*V* = 1.75·(Δ*T*)^1.41^; *R*² = 0.660; *P *<* *0.001; Fig. 6b), with significantly different slope from velocities calculated from acoustic values (*P *<* *0.0001) or from debarked samples (*P *<* *0.0001). For ∆*T* = 6 K, we observed an acoustic propagation rate of 36 mm s^−1^, whereas propagation monitored with infrared thermography was measured at 21 mm s^−1^.

### Experiments with other species

**Figure 7:**
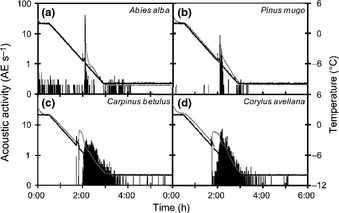
Typical acoustic activity (acoustic emissions s^−1^) close to the ice nucleation point in the wood of different species: (a) *Abies alba*, (b) *Pinus mugo*, (c) *Carpinus betulus* and (d) *Corylus avellana* during freezing. Black lines represent the air temperature of the cooling chamber, and grey lines represent the temperature of the wood.

As in *P. abies*, two phases of AEs activity during freezing were observed in *Abies alba* and *P. mugo* (Fig. 7a,b), with higher activity at the time of nucleation (10–50 AE s^−1^). Two phases with similar intensity also were observed in the angiosperms *Carpinus betulus* and *Coryllus avellana* (Fig. 7c,d); they emitted overall more AEs than conifers.

## Discussion

During freeze–thaw cycles in plant xylem, previous studies have demonstrated AE activity on freezing, whereby the onset of AEs exactly corresponded to the time the freezing exotherm was registered (Mayr *et al*., 2007; Charrier *et al*., 2014a). In previous studies, AEs were recorded at one location per sample (Mayr & Zublasing, 2010), which did not allow us to locate AE sources. Here, several sensors were placed along the sample (Fig. 1) and, in addition, the point of ice nucleation was controlled (Fig. 2). As in former studies, AEs were recorded after the onset of freezing, but observed AE patterns were both temporally and spatially more complex than hypothesized: AE activities differed significantly along the sample during ice nucleation and propagation. Close to the ice nucleation point, many AEs were generated at the time of ice nucleation (phase I; Fig. 3a,f,g), but the number of AEs decreased with the distance to the nucleation point (Figs 3c–e,h, 4c; Table 1). A second phase (phase II) in acoustic activity was observed when xylem temperatures proceeded to decrease. These two phases indicate a two‐step process leading to AEs: during ice nucleation and propagation, and during temperature decrease, once exothermal heat is dissipated.

When water molecules turn from liquid into ice, the structure becomes more stable, which releases energy (latent heat = 334 J g^−1^). This energy is recorded as an increase in temperature (exotherm; Muldrew *et al*., 2004). In our experiments, the exotherm was recorded by thermocouples and by infrared thermography (Fig. 2). This method enabled us to estimate the ice propagation velocity, which was *c*. 16 mm s^−1^ when nucleation occurred at *c*. −5°C. According to Hacker & Neuner (2008) and Pramsohler *et al*. (2012), the ice propagation started from the nucleation point and spread along the sample, with higher longitudinal than radial speed. According to Langer *et al*. (1978) and Rauschenberger *et al*. (2013) the degree of supercooling influences the ice propagation velocity. As in water‐filled capillary tubes (Teraoka *et al*., 2002; Ribeiro *et al*., 2006), the velocity increased with ∆*T* following a power‐law in the present experiments on stem samples (Fig. 6b). During phase I, the time shift in AE peak activity indicated *c*. 30 mm s^−1^ propagation velocity, similar to the dendritic growth rate of ice crystals in an homogeneous medium (e.g. water‐filled capillary tube: 31 mm s^−1^ at ∆*T* = 5 K; Ribeiro *et al*., 2006), but 2× faster than detected by infrared thermography (Fig. 6b). The latter technique was based on the increase in surface temperature, which is probably smoothed and delayed by the radial diffusion of heat through the bark. Furthermore, the ice front can propagate only after exothermal heat diffused within the sample (Shibkov *et al*., 2003, 2005). Accordingly, in debarked samples, faster and more accurate ice propagation was observed (21 mm s^−1^), closer to values calculated from acoustic signals.

Several hundred AEs were detected within 10 s (10–50 AE s^−1^) after exotherm formation on the sensors close to the nucleation point (Fig. 3a,f,g), but fewer AEs were recorded on sensors located at greater distances, even when ice propagated (Fig. 3c–e,h). This pattern during phase I was not caused by the artificial nucleation as acoustic activity was similar after spontaneous nucleation (Fig. 5a,b). Also, removal of the bark did not influence AE patterns (Fig. 5e). We suggest that AEs detected during phase I were generated by the propagating ice front because the time difference of peak AE activity between sensors (several seconds) is much greater than the sound propagation of acoustic waves in wood and ice (*c*. 0.03 ms; Charrier *et al*., 2014b). The propagation velocity of the AE source during phase I was calculated as the distance between two acoustic sensors divided by the time difference of mean peak AE activity during phase I. We used this calculation because of the extreme difference in number of AE along the sample and the enormous shift in attenuation between liquid and frozen sap (Charrier *et al*., 2014b). Triangulation methods were therefore not adopted as one AE could not clearly be identified on at least three sensors. However, the calculated ice propagation velocities exhibited a similar temperature dependence to those in water‐filled capillary tubes (Ribeiro *et al*., 2006) and was similar to the speed of ice propagation observed via infrared thermography directly on wood (Fig. 6b). This would indicate that ice propagation along the water column was not delayed by wood anatomy (i.e. pits) in the longitudinal direction (Hacker & Neuner, 2007; Neuner *et al*., 2010).

Along the sample, the decreasing number of AEs detected during ice propagation is most likely based on the attenuation properties of the sample. Due to the much lower attenuation in frozen vs unfrozen wood (Charrier *et al*., 2014b), AEs could be recorded mainly along the frozen section of the sample, whereas AEs were strongly attenuated in the remaining unfrozen part. The propagating ice thus caused decreasing attenuation in the sample so that distally originated signals could be detected by all sensors.

After the temperature reached its maximum during exotherm formation (−1 to −2°C) and started to decrease again, phase II of acoustic activity was observed in all sensors and lasted until temperatures stabilized (−10°C). These patterns were also observed in *P. mugo* (Fig. 7a) and *A. alba* (Fig. 7b). Interestingly, phase II was comparably more pronounced in the studied angiosperms *C. betulus* (Fig. 7c) and *C. avellana* (Fig. 7d). AEs detected in angiosperms during a freeze–thaw cycle have been partially related to cavitation in living cells, which constitute higher proportion of xylem tissue than in conifers, (Kikuta, 2003; Kikuta & Richter, 2003; Charrier *et al*., 2014a; Kasuga *et al*., 2015).

Previous studies have already demonstrated that decreasing temperatures after ice formation lead to AEs (Mayr & Zublasing, 2010; Charrier *et al*., 2014a). Presented experiments now reveal that the temperature at which ice nucleation occurs, influences phase I of AEs. What are the possible underlying mechanisms for this temperature dependence? At negative temperatures, water remains liquid until ice nucleates. This metastable state, called supercooling, is common in plant tissues and can reach −38°C (Fujikawa & Kuroda, 2000). When supercooled water freezes, a fraction instantaneously turns into ice (Chevalier *et al*., 2000; Otero & Sanz, 2000). The ratio can be calculated according to heat balance:

(Eqn 1) $$m_{w}\cdot Cp_{w}\cdot \Delta T=L\cdot m_{i}$$

(*m*_w_ and *m*_i_, mass of liquid and ice, respectively; *Cp*_w_, specific heat capacity of liquid water; ΔT, degree of supercooling; *L,* latent heat of water). According to Eqn 1, at the onset of freezing, the ratio of frozen to unfrozen volume is:

(Eqn 2) $$\frac{m_{i}}{m_{w}}=\frac{Cp_{w}\cdot \Delta T}{L}$$

with *L* = 334 kJ kg^−1^ and *Cp*_w_ = 4.18 kJ kg^−1^ K^−1^ at 25°C (Weast, 1984).

In our experiments, the instantaneous ice ratio was between 3% at −2.5°C and 8% at −6.5°C (according to Schlüter *et al*., 2004). If we consider that low Ψ_ice_ is the driving force inducing water flows in wood (Améglio *et al*., 2001), liquid water is pulled to the nucleation point, increasing the tension up to the threshold of cavitation and thus generating AEs of stronger energy (Mayr *et al*., 2007; Mayr & Zublasing, 2010; Mayr & Rosner, 2011; Ponomarenko *et al*., 2014). At the ice–liquid interface, temperature has a strong influence on the tension induced by ice (*c*. −1 MPa K^−1^; Hansen & Beck, 1988; Cavender‐Bares, 2005). Accordingly, when ice nucleated at low supercooling (2.5 K), the instantaneous ice proportion was low (cf. Eqn 2) and the increase in tension small (Hansen & Beck, 1988), which caused only few AEs of low energy (Fig. 6a). At moderate initial Ψ, the tension induced by ice was probably too low to generate AEs (Fig. 5c; also see Mayr *et al*., 2007; Mayr & Sperry, 2010). At the ice–liquid interface, an increase in concentration of osmolytes may further increase the tension (Sevanto *et al*., 2012). In our experimental conditions, the concentration should have increased from *c*. 6% at 2.5 K to *c*. 18% at 6.5 K, which should have generated an increase in osmotic pressure from 0.1 (2.5 K) to 0.4 kPa (6.5 K). This effect is minor compared with the Ψ induced by ice. According to Saclier *et al*. (2010), ultrasounds may induce the formation of cavitating bubbles within water, which may therefore become ice nucleation sites. Thus, a chain reaction might be initiated when the first crystal is formed, generating ultrasound that induces other bubbles to cavitate; the more metastable the liquid, the less energy would be needed for this hypothetical mechanism to take place.

The decrease in density of ice with respect to liquid water (917 vs 1000 kg m^3^ at 0°C) induces *c*. 9% increase in local pressure at the ice–liquid interface (Hare & Sorensen, 1987; Holten *et al*., 2012). Furthermore, heat released by ice crystallization increased the temperature of samples and thus caused lower tension at the ice–liquid interface (see earlier). In Figs 2 and 3, maximal temperatures on the wood during exotherm formation were equal to −1.6 ± 0.14°C, close to the melting point of ice. This combined effect of increased pressure and temperature may have compensated the tension induced by the ice nucleation and caused a break in AE generation. When water freezes, not all of the water molecules are included in the ice lattice and solutes are expelled (Sevanto *et al*., 2012); thus, around the ice, a layer of liquid water remains with high concentrations of solutes. When the temperature decreases further, the volume of ice increases, whereas the thickness of the liquid layer decreases and solute concentration increases, inducing a second phase in AE generation. When most of the water was frozen, the exothermal heat dissipated and the sample temperature and Ψ_ice_ decreased. Subsequently, AEs were generated again, until the temperature stabilized. On thawing, tensions decreased, which did not induce AEs.

## Conclusion

We identified two different phases of freezing‐induced AEs, separated by an acoustically inactive period. Based on the chemical potential of ice and on the temperature at the ice–liquid interface, ice nucleation and propagation cause tensions in the xylem sap high enough to generate cavitation events and, consequently, a first phase of AEs. Exothermal heat from ice formation and increase in pressure due to volume changes subsequently release the tension induced by ice. In phase II, when temperatures decrease again, the chemical potential of ice increases the tension and generates new cavitation events and AEs in the remaining liquid layer. These results indicate that cavitation occurs at the ice nucleation point, the AE source probably moves with the propagating ice front, through a complex process influenced by several factors (e.g. degree of supercooling, balance between water potential of ice and sap); the ice nucleation temperature influences phase I, whereas Ψ influences both phases. These results highlight the complexity of freezing in heterogenous tissues (especially the balance between the exothermal heat in combination with decreasing temperature). Finally, we demonstrated that acoustic detection might be useful for localizing ice nucleation and the propagation front within wood and that propagation along the water column is not delayed by anatomy. Acoustic emission techniques would therefore warrant use on more complex tree architectures (e.g. ramified branches and/or large trunks) and potentially *in natura*.
